# Handwashing practices and associated factors among public primary schoolchildren in the Benadir Region, Somalia

**DOI:** 10.3389/fpubh.2026.1958243

**Published:** 2026-09-16

**Authors:** Abdinasir Bashir Nuh, Ali Bile Farah, Abdifatah Afyare, Abdirashid Hassan Da’ar, Mohamed Yusuf Abdi

**Affiliations:** 1 Faculty of Medicine and Health Sciences, University of Health Sciences, Bosaso, Puntland, Somalia; 2 Department of Politics and IR, Faculty of Social Science, Salaam University, Mogadishu, Somalia; 3 Head of the Laboratory Department, Shaafi Hospital, Mogadishu, Somalia; 4 Department of Microbiology and Laboratory Sciences, Faculty of Medicine and Health Sciences, SIMAD University, Mogadishu, Somalia

**Keywords:** handwashing practice, hygiene behavior, primary schools, schoolchildren, Somalia, wash

## Abstract

**Background:**

In Somalia, numerous schools lack adequate water, sanitation, and hygiene (WASH) facilities, which is associated with a high burden of diarrheal and respiratory infections among school-aged children. Despite this, there is a paucity of data on handwashing practices and their associated factors, particularly in the Benadir Region. This study evaluated handwashing practices and identified factors associated with proper handwashing among primary schoolchildren in the Benadir Region of Somalia.

**Methods:**

A cross-sectional study was conducted in schools From January 1 to January 31, 2026, employing a multistage cluster sampling technique. The study involved 834 students in grades 5–8, chosen from five public primary schools. Trained interviewers collected data using structured, pretested questionnaires. The data were inputted and analyzed using SPSS version 27. To determine the factors associated with effective handwashing practices, multivariable logistic regression analysis was performed, with statistical significance defined as *p* < 0.05.

**Results:**

Overall, 76.0% (95% CI: 73.1–78.8) of the students reported practicing proper handwashing. In the multivariable analysis, several factors were independently associated with proper handwashing. These included recognizing odor elimination as a benefit of handwashing (AOR = 3.16, 95% CI: 2.03–4.94), having parents (AOR = 3.59, 95% CI: 1.55–8.29) or teachers (AOR = 3.06, 95% CI: 1.27–7.37) as the main influence for handwashing, having a handwashing station (AOR = 1.53, 95% CI: 1.02–2.29), having handwashing facilities at school (AOR = 2.12, 95% CI: 1.27–3.54), and the school celebrating Global Handwashing Day (AOR = 2.42, 95% CI: 1.12–5.23).

**Conclusion:**

Approximately 75% of primary school students reported washing their hands properly. Proper handwashing was significantly associated with perceived personal benefits, positive social influences, and school WASH infrastructure. Strengthening school hygiene programs, leveraging positive social influences, and upgrading WASH infrastructure may support higher handwashing compliance among primary school students.

## 1 Introduction

Handwashing with soap entails physically cleaning hands of dirt, organic matter, and dangerous microorganisms using soap and water. This approach is widely recognized as one of the most economical and successful public health strategies for stopping the spread of infectious diseases in community settings (1–4). Global initiatives, such as Global Handwashing Day, aim to increase awareness of hand hygiene and promote washing hands with soap at crucial times to prevent the transmission of infectious diseases (5). School-age children in many developing countries frequently do not understand the significance of washing their hands before meals, after using the restroom, and before cooking (6, 7). Crowded conditions in schools and childcare facilities increase the risk of spreading infectious diseases. Alarmingly, fewer than 5% of people in these areas consistently practice handwashing (8). Improving children’s health requires the prevention of infectious diseases, particularly diarrheal diseases and acute respiratory infections (9, 10). Poor handwashing habits have led to approximately 400 million children contracting worm infections each year (11). An estimated 443 million school days are lost each year due to water-related illnesses, significantly affecting student attendance in developing countries (5). Despite the introduction of national initiatives, such as the School WASH Programme, to enhance hygiene habits, there is still an urgent need for effective and lasting strategies to promote handwashing among school-aged children (12). Moreover, numerous schools in Somalia encounter difficulties due to inadequate water supply, scarce soap availability, and poor sanitation infrastructure, which can impede students’ adoption of proper handwashing habits (13). These issues are further exacerbated by the limited data on handwashing practices among school children. Additionally, the absence of regular monitoring and lack of adequate handwashing facilities in many schools heighten the risk of disease transmission among students. Although hand hygiene is crucial for curbing the spread of infectious diseases, evidence regarding handwashing practices and the factors that influence them among Somali schoolchildren is scarce, particularly in the Benadir Region. This study sought to assess handwashing practices and the factors associated with proper handwashing among public primary schoolchildren in the Benadir Region, Somalia.

## 2 Materials and methods

### 2.1 Study setting, design, and period

A cross-sectional study was conducted in public primary schools in the Benadir Region of Mogadishu, Somalia, from 1 to January 31, 2026. The Benadir Region has 17 districts and had 41 public primary schools during the 2025/2026 academic year, all of which were included in this study.

### 2.2 Source and study population

All students attending public primary schools in the Benadir Region during the 2025/2026 academic year constituted the source population. The research specifically targeted students in grades 5–8 who were present in the chosen schools at the time of data collection.

### 2.3 Inclusion and exclusion criteria

During the data collection period, all students from grades 5 to 8 who were enrolled in and attending the selected schools were considered for inclusion. However, students who were very ill or unable to communicate well, even with support, were excluded.

### 2.4 Sample size determination

The required sample size was determined using a formula for a single population proportion, considering a previous prevalence rate of *p* = 57.6%. This percentage represents the proportion of Ethiopian primary school students who practice proper handwashing (14).

$$n_{0}=\frac{{\left(Z_{\alpha /2}\right)}^{2}\times \rho (1-\rho )}{d^{2}}$$

At a 95% confidence level, *Z* is valued at 1.96, while N represents the required minimum sample size, with a 5% margin of error. This study considered two design effects. After adjusting for a 10% non-response rate, the total sample size for this study was 834.

### 2.5 Sampling procedure and technique

A multi-stage sampling procedure was used to select study participants. In the first stage, 5 public primary schools were selected via simple random sampling from the 41 public primary schools operating in the Benadir Region during the 2025/2026 academic year: Towfiiq, Boqolsoon, Mahamuud Warsame Siyo, Yasiin Cusman, and 21st November. In the second stage, the total eligible population of students in Grades 5–8 across the five selected schools was determined ( N=2,312 ). The required sample size ( n=834 ) was proportionally allocated to each school based on its population size using the probability proportional to size (PPS) formula:

$$n_{i}=(\frac{N_{i}}{N})\times n$$

where $n_{i}$ is the sample size allocated to school i , $N_{i}$ is the number of eligible students in school i , N is the total eligible student population across all five schools ( 2,312 ), and n is the total target sample size ( 834 ). Accordingly, sample allocations were: Towfiiq ( n=212 ), Boqolsoon ( n=35 ), Mahamuud Warsame Siyo ( n=151 ), Yasiin Cusman ( n=228 ), and 21st November ( n=208 ). In the third stage, the allocated sample per school was proportionally divided across Grades 5 through 8 based on grade-level enrollment. Finally, individual students were selected from official classroom attendance rosters as sampling frames using the lottery method for simple random sampling to ensure equal selection probability for every student (Figure 1).

**Figure 1:**
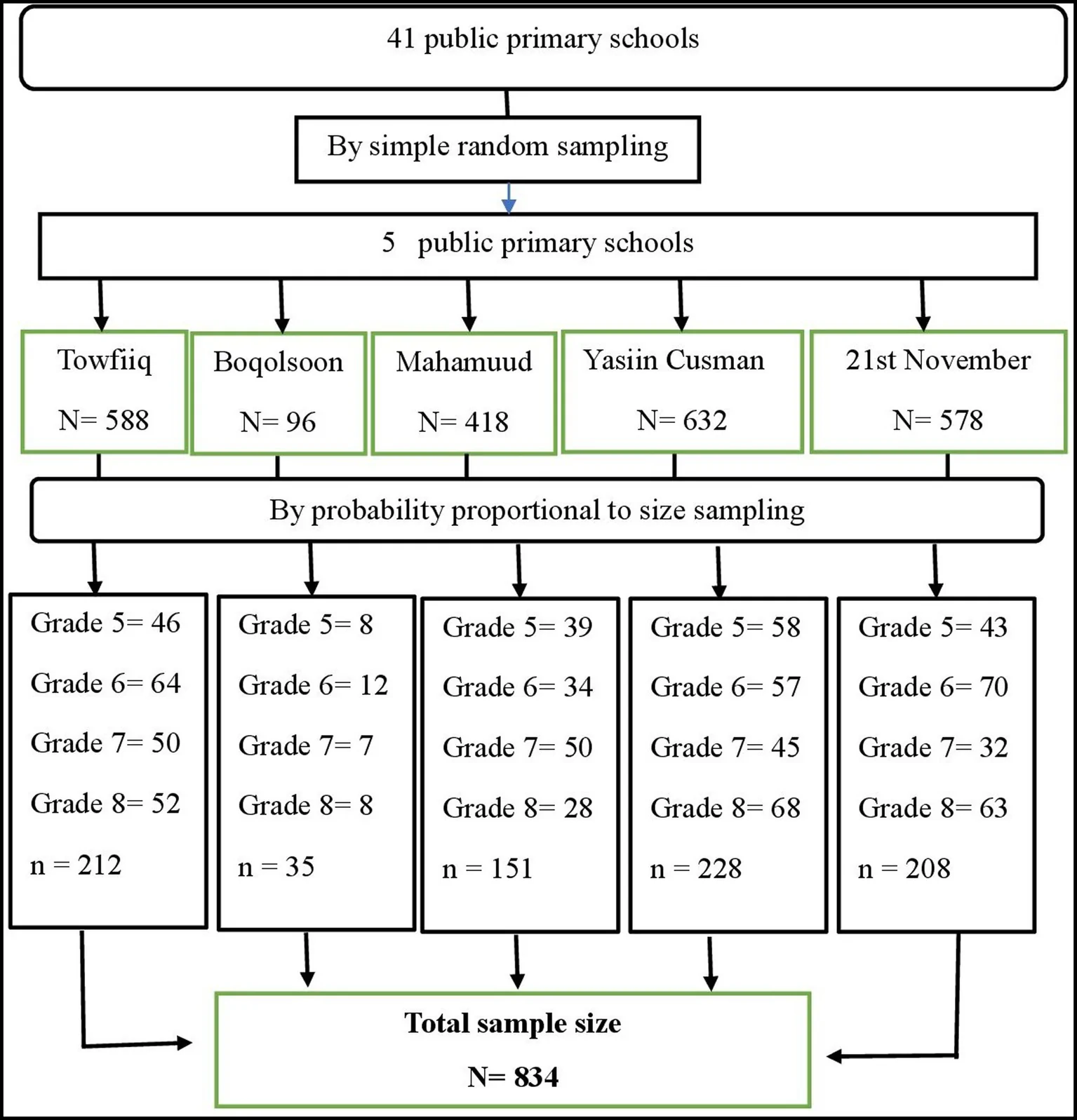
Multistage sampling procedure for selecting study participants in the Benadir Region, Somalia.

### 2.6 Data collection methods

Data collection was conducted using a structured questionnaire that had been pretested and administered by trained interviewers who had received training. This questionnaire, originally in English and adapted from previous studies (14, 15), was translated into Somali. Public health experts checked its content validity, and it was pretested before the main data collection. The questionnaire comprised four sections: (1) sociodemographic information of students and parents, (2) handwashing habits, (3) personal and social factors influencing handwashing, and (4) handwashing facilities at schools. Handwashing practices were assessed using six behavior-related questions. Responses that conformed to the recommended handwashing practices were assigned a score of 1, while all other responses received a score of 0. Specifically, participants who reported washing their hands in the past 12 h (1 = Yes, 0 = No). Usual handwashing time was considered (1 = before meals or after toilet, 0 = after meals, after work, or after play). The Item used for handwashing (1 = water and soap; 0 = water only or water with soil). Duration of handwashing at a time (1 = 20 s to 1 min, 0 = <20 s or do not know). For the frequency of soap use before meals, responses were (1 = always or often, 0 = sometimes or never). The frequency of soap use after toilet use (1 = always or often, 0 = sometimes or never). The individual item scores were aggregated to construct an overall composite handwashing score ranging from 0 to 6. The internal consistency and reliability of the composite scale were confirmed in the study population. Based on a pre-specified cutoff point, participants with scores between 4 and 6 ( ≥66.7% ) were classified as having proper handwashing practices, while those with scores between 0 and 3 (≤50%) were classified as having improper handwashing practices. Trained public health graduates collected the data under the supervision of the principal investigator. Prior to commencement, both data collectors and supervisors participated in a two-day training session that covered the study’s objectives, ethical considerations, interviewing techniques, and study procedures. A pre-test was conducted on 5% of the total sample at a public primary school, which was comparable to the study setting, prior to the main investigation. The pre-test results led to small changes to make the questionnaire clearer and better-organized. During data collection, the completed questionnaires were checked daily to ensure that they were complete and consistent.

### 2.7 Study variables

Dependent Variable

- Handwashing practice

Independent Variables

- Sociodemographic details (such as age, gender, grade level, parental education, and occupation).
- Personal and social factors (perceived benefits of handwashing, reasons for not washing hands, sources of information, and role models influencing handwashing behavior).
- Factors at the facility level, such as accessibility and availability of water, soap, and handwashing stations.

### 2.8 Data processing and analysis

Data analysis was conducted using IBM SPSS Statistics (version 27) after ensuring that the data were complete and transferred to Microsoft Excel 2019. Descriptive statistics were presented through frequencies and percentages. To identify the factors associated with proper handwashing practices, crude odds ratios (CORs) and their 95% confidence intervals (CIs) were calculated using bivariate logistic regression. Variables with a *p*-value below 0.20 were included in a multivariable logistic regression model. Adjusted odds ratios (AORs) with 95% confidence intervals were computed, with statistical significance set at *p* < 0.05.

### 2.9 Operational definitions

Handwashing facility: Students noted that there is a specific area in the school for handwashing.

Handwashing station: During the survey, students reported that the handwashing facility was functioning, regardless of the availability of soap.

Availability of soap and water: During the survey, both soap and water were available at the school’s handwashing station.

Referents for handwashing practice: The students identified the person who most influenced their handwashing behavior, such as parents, teachers, health professionals, or friends.

Benefits of handwashing: The students’ primary perceived reasons for handwashing were disease prevention, hand cleanliness, and elimination of unpleasant odors.

Reasons for not washing hands: The most common self-reported reasons for not washing hands were forgetfulness, laziness, lack of time, lack of interest, lack of soap, and lack of water availability.

Source of handwashing information: Television, radio, leaflets, newspapers, mini-media, and textbooks served as the main sources of information for the students regarding handwashing.

Global Handwashing Day celebration: The student’s self-reported awareness of the school’s organization of activities to observe Global Handwashing Day.

### 2.10 Ethical considerations

This study was approved by the Ethical Review Committee (ERC) of the University of Health Sciences (Ref No: UOHS/ERC/2025/0146). Written informed consent was obtained from the parents or legal guardians of the participants, and participation was completely voluntary.

## 3 Results

### 3.1 The participants’ sociodemographic characteristics

In this study, 834 students were involved, resulting in a 100% response rate. The participants included 188 students (22.5%) from Grade 5, 236 (28.3%) from Grade 6, 213 (25.5%) from Grade 7, and 197 (23.6%) from Grade 8. Of these students, 462 (55.4%) were female, and 427 (51.2%) were 14 years or older. Regarding parental education, 518 mothers (62.1%) and 658 fathers (78.9%) had formal schooling. Nearly half of the mothers 403 (48.3%) were housewives. Among the fathers, 335 (40.2%) were employed, 272 (32.6%) were self-employed, and 227 (27.2%) were unemployed (Table 1).

**Table 1:** Sociodemographic characteristics of primary schoolchildren in the Benadir Region, Somalia.

Variables	Categories	Frequency	Percent
Grade	Grade 5	188	22.5
	Grade 6	236	28.3
	Grade 7	213	25.5
	Grade 8	197	23.6
Age	<14	407	48.8
	≥14	427	51.2
Sex	Male	372	44.6
	Female	462	55.4
Education of Mother	Non-Formal Education	316	37.9
	Formal Education	518	62.1
Occupation of Mother	Housewife	403	48.3
	Non-Housewife	431	51.7
Education of Father	Non-Formal Education	176	21.1
	Formal Education	658	78.9
Occupation of Father	Employed	335	40.2
	Unemployed	227	27.2
	Self-employed	272	32.6

### 3.2 Handwashing practice among students

Among the 834 participants, 76.0% (634) followed the proper handwashing practices. On the day of the interview, 93% (n = 776) of the participants reported washing their hands in the last 12 h. The most common occasions for handwashing were before meals (60.6%) and after toilet use (17.3%). Regarding the materials used, 59.6% used water and soap, 29.3% used only water, and 11.2% used water with soil. Handwashing lasted less than 20 s for 25.4% of participants, between 20 s and 1 min for 25.7, and 48.9% did not know the recommended duration. Soap was consistently used before meals by 37.2% of the participants, whereas 14.4% never used soap. After using the toilet, 50.6% always used soap, whereas 9.8% never did (Table 2).

**Table 2:** Handwashing practices of primary schoolchildren in the Benadir Region, Somalia.

Variables	Categories	Frequency	Percent
Have you performed handwashing within the past 12 h?	Yes	776	93.0
	No	58	7.0
Usual handwashing time	Before meal	505	60.6
	After meal	111	13.3
	After work	27	3.2
	After play	47	5.6
	After toilet	144	17.3
Item used for handwashing	Water only	244	29.3
	Water and Soap	497	59.6
	Water with soil	93	11.2
Duration of handwashing at a time	<20s	212	25.4
	20s-1min	214	25.7
	Do not know	408	48.9
How frequently do you use soap to wash your hands before eating?	Always	310	37.2
	Often	94	11.3
	Sometimes	310	37.2
	Never	120	14.4
How often do you use soap to wash your hands after using the toilet?	Always	422	50.6
	Often	53	6.4
	Sometimes	277	33.2
	Never	82	9.8
Overall practice			
	Proper	634	76%
	Improper	200	24%

### 3.3 Personal and social influences for handwashing practices

Almost half of those surveyed (46.8%) viewed handwashing as a way to prevent illness, while 33% saw it as a means to clean their hands, and 20.3% believed it was a way to eliminate odors. The most common reason for neglecting handwashing was forgetfulness (43.4%), followed by lack of water (20.3%), laziness (9.6%), lack of interest (9.0%), no soap available (9.0%), and insufficient time (8.8%). The main sources of information about handwashing were textbooks (40.3%) and television (34.7%), with minor contributions from mini media (9.1%), radio (7.3%), newspapers (6.4%), and leaflets (2.3%). In terms of influence, parents were the most significant figures (60.7%), followed by teachers (21.3%), health professionals (14.0%), and friends (4.0%) (Table 3).

**Table 3:** Personal and social influences for handwashing practices among primary schoolchildren in the Benadir Region, Somalia.

Variables	Categories	Frequency	Percent
Benefit of handwashing			
	To prevent disease	390	46.8
	To eliminate unpleasant odors	169	20.3
	To clean hand	275	33.0
What is the primary reason for not washing your hands?			
	Forgetfulness	362	43.4
	Laziness	80	9.6
	Lack of time	73	8.8
	Lack of interest	75	9.0
	Lack of water	169	20.3
	Lack of soap	75	9.0
Source of media information on handwashing practices			
	Television	289	34.7
	Radio	61	7.3
	Leaflets	19	2.3
	Newspaper	53	6.4
	Mini-media	76	9.1
	Textbooks	336	40.3
Referents for handwashing practice			
	Parents	506	60.7
	Teachers	178	21.3
	Health professionals	117	14.0
	Friends	33	4.0

### 3.4 Availability of handwashing facilities

Most students (87.5%) reported that their schools had handwashing facilities, while 12.5% did not. Of these facilities, 58.0% were easy to reach on foot and 42.0% were near restrooms. About 65.3% of students were aware of the handwashing stations, yet just 32.7% reported that soap and water were accessible for handwashing at their school. In terms of awareness, 17.7% of schools took part in Global Handwashing Day, whereas 82.3% did not participate (Table 4).

**Table 4:** Availability of handwashing facilities in selected primary schoolchildren in the Benadir Region, Somalia.

Variables	Categories	Frequency	Percent
Is there handwashing facility in school	Yes	730	87.5
	No	104	12.5
Location of handwashing facilities	Next to latrines	350	42
	Within walking distance	484	58
Presence of handwashing station	Yes	545	65.3
	No	289	34.7
Availability of Soap and water	Yes	273	32.7
	No	561	67.3
Does the school celebrate Global Handwashing Day?	Yes	148	17.7
	No	686	82.3

### 3.5 Factors associated with hand washing practice

In the bivariable logistic regression analysis, several factors were significantly associated with proper handwashing practices. These factors included students’ grade level, perceived benefits of handwashing, reasons for not washing hands, referents for handwashing practice, presence of a handwashing station, availability of handwashing facilities, availability of soap and water, and celebration of Global Handwashing Day (*p* < 0.05). Age and father’s occupation met the inclusion criteria (*p* < 0.20) and were thus incorporated into the multivariable logistic regression analysis. However, no significant correlation was found between appropriate handwashing practice and sex, mother’s education, mother’s work, father’s education, or the source of media knowledge about handwashing (*p* > 0.20). The multivariable logistic regression analysis showed that students who identified the removal of unpleasant odors as a benefit of handwashing were more likely to practice proper handwashing than those who saw illness prevention as the main benefit (AOR = 3.16, 95% CI: 2.03–4.94, *p* < 0.001). Students reported a higher likelihood of washing their hands correctly if their schools provided a handwashing station compared to those without such facilities (AOR = 1.53, 95% CI: 1.02–2.29, *p* = 0.041). Similarly, students who reported having handwashing facilities at their schools had higher odds of proper handwashing practice than those without such facilities (AOR = 2.12, 95% CI: 1.27–3.54, *p* = 0.004). Students from schools that celebrated Global Handwashing Day were more likely to wash their hands correctly than those from schools that did not participate in the event (AOR = 2.42, 95% CI: 1.12–5.23, *p* = 0.025). Additionally, students who identified parents (AOR = 3.59, 95% CI: 1.55–8.29, *p* = 0.003) or teachers (AOR = 3.06, 95% CI: 1.27–7.37, *p* = 0.013) as their primary referents had higher odds of proper handwashing than those who identified friends as their primary referents. After adjustment, grade level, age, father’s occupation, reasons for not washing hands, availability of soap and water, and health professionals as referents were not significantly associated with proper handwashing practice (*p* > 0.05) (Tables 5, 6).

**Table 5:** Analysis of bivariable logistic regression on factors associated proper handwashing practices among primary schoolchildren in the Benadir Region, Somalia.

Variable	Response	Handwashing practice		COR (95% CI)	*p*-value
		Proper	Improper		
Grade	Grade 5	135	53	1	1
	Grade 6	191	45	1.25 (0.81–1.94)	0.307
	Grade 7	176	37	2.09 (1.35–3.25)	0.001
	Grade 8	132	65	2.34 (1.48–3.72)	0.001
Age	<14 years	321	86	1	1
	≥14 years	313	114	0.74 (0.53–1.01)	0.060
Sex	Male	290	82	1	1
	Female	344	118	1.21 (0.88–1.67)	0.240
Mother’s Education	Non-formal education	241	75	1	1
	Formal education	393	125	1.02 (0.74–1.42)	0.896
Mother’s Occupation	Housewife	314	89	1	1
	Non-housewife	320	111	1.22 (0.89–1.68)	0.215
Father’s Education	Non-formal education	134	42	1	1
	Formal education	500	158	1.01 (0.68–1.49)	0.967
Father’s Occupation	Employed	261	74	1	1
	Unemployed	163	64	1.04 (0.71–1.53)	0.836
	Self-employed	210	62	0.75 (0.50–1.13)	0.167
Benefit of handwashing	To prevent disease	339	51	1	1
	To eliminate unpleasant odors	108	61	3.13 (2.12–4.61)	0.001
	To clean hands	187	88	0.83 (0.56–1.25)	0.375
Reasons for not washing hands	Forgetfulness	308	54	1	1
	Laziness	56	24	3.03 (1.74–5.28)	0.001
	Lack of time	45	28	1.24 (0.63–2.43)	0.535
	Lack of interest	43	32	0.85 (0.44–1.67)	0.641
	Lack of water	133	36	0.71 (0.37–1.38)	0.315
	Lack of soap	49	26	1.96 (1.07–3.58)	0.028
Source of media information about handwashing	Television	218	71	0.88 (0.61–1.28)	0.508
	Radio	46	15	0.88 (0.47–1.67)	0.697
	Leaflets	14	5	0.81 (0.28–2.31)	0.686
	Newspaper	39	14	0.80 (0.41–1.55)	0.510
	Mini-media	56	20	0.81 (0.45–1.43)	0.456
	Textbooks	261	75	1	1
Referents for handwashing practice	Parents	416	90	6.27 (3.03–12.98)	0.001
	Teachers	131	47	3.78 (1.76–8.14)	0.001
	Health professionals	73	44	2.25 (1.03–4.94)	0.043
	Friends	14	19	1	1
Presence of handwashing station	No	184	105	1	1
	Yes	450	95	2.70 (1.95–3.75)	0.001
Handwashing facility in school	No	56	48	1	1
	Yes	578	152	3.26 (2.13–4.99)	0.001
Availability of soap and water	No	392	169	1	
	Yes	242	31	3.37 (2.22–5.10)	0.001
Celebrate Handwashing Day	No	497	189	1	1
	Yes	137	11	4.74 (2.51–8.95)	<0.001

COR, crude odds ratio; CI, confidence interval.

**Table 6:** Analysis of multivariable logistic regression on factors associated proper handwashing practices among primary schoolchildren in the Benadir Region, Somalia.

Variable	Response	AOR (95% CI)	*p*-value
Grade	Grade 5	1	1
	Grade 6	1.08 (0.63–1.88)	0.773
	Grade 7	1.65 (0.97–2.80)	0.066
	Grade 8	1.62 (0.95–2.77)	0.076
Age	<14 years	1	1
	≥14 years	1.26 (0.84–1.87)	0.259
Father’s Occupation	Employed	1	1
	Unemployed	0.85 (0.54–1.34)	0.483
	Self-employed	1.00 (0.62–1.60)	0.990
Benefit of handwashing	To prevent disease	1	
	To eliminate unpleasant odors	3.16 (2.03–4.94)	<0.001*
	To clean hands	1.52 (0.93–2.48)	0.093
Reasons for not washing hands	Forgetfulness	1	
	Laziness	1.65 (0.87–3.16)	0.128
	Lack of time	0.76 (0.35–1.64)	0.480
	Lack of interest	0.57 (0.26–1.25)	0.161
	Lack of water	0.53 (0.25–1.13)	0.099
	Lack of soap	1.56 (0.79–3.10)	0.200
Presence of handwashing station	No	1	1
	Yes	1.53 (1.02–2.29)	0.041*
Handwashing facility in school	No	1	
	Yes	2.12 (1.27–3.54)	0.004*
School celebrate handwashing day	No	1	
	Yes	2.42 (1.12–5.23)	0.025*
Availability of soap and water	No	1	
	Yes	1.53 (0.90–2.62)	0.119
Referents for handwashing practice	Parents	3.59 (1.55–8.29)	0.003*
	Teachers	3.06 (1.27–7.37)	0.013*
	Health professionals	2.00 (0.82–4.88)	0.130
	Friends	1	1

AOR, adjusted odds ratio; CI, confidence interval. *Statistically significant at *p* < 0.05.

## 4 Discussion

Handwashing is an essential practice for maintaining hygiene and preventing the transmission of infectious diseases. Educational institutions play a significant role in inculcating this habit in children from an early age. In the present study, 76.0% (95% CI: 73.1–78.8) of primary school children in the Benadir Region of Somalia demonstrated proper handwashing practices. This prevalence was substantially higher than those reported in previous studies from Ethiopia, including Wolaita Zone (28.1%) (15), Arba Minch Town (22.3%) (16), and Sebeta Town (32.8%) (17). It was also higher than the prevalence reported in studies conducted in Colombia (33.6%) (18), India (44.2%) (19), and Wuhan, China (42.1%) (20). However, this is still lower than the 83.6% rate among Saudi Arabian primary school students in Riyadh (21). These discrepancies likely reflect variations in study populations, school infrastructure, and socioeconomic contexts. For example, higher rates in Saudi Arabia may be related to greater baseline access to continuous running water and soap. Additionally, differences in measurement methods, such as self-reported questionnaires versus direct observational audits, may contribute to these observed discrepancies. The significance of personal motivation in adhering to handwashing practices was evident in the fact that students who believed that “removing bad smells” was the primary benefit of handwashing were more than three times more likely to wash their hands correctly than those who believed that “preventing disease” was the primary benefit. This suggests that immediate sensory cues, such as odor elimination and soap fragrance, correlate more strongly with children’s practices than abstract health concepts, such as germ theory or long-term disease prevention. Our findings are in accordance with research from Ghana, which observed that schoolchildren’s hygiene practices were more strongly correlated with sensory cues and disgust than with health knowledge alone (22). In a similar vein, a study conducted in Eastern Ethiopia discovered that although handwashing knowledge was high, actual practice was low, indicating a gap between knowledge and action that is frequently filled by immediate emotional or sensory signals rather than health education alone (23). The physical environment of schools was significantly associated with student handwashing practices. In this study, students attending schools equipped with dedicated handwashing facilities demonstrated more than two-fold higher odds of maintaining proper hygiene. This result is consistent with a study conducted in Eastern Ethiopia that found a significant relationship between the availability of handwashing stations and proper handwashing practices among primary schoolchildren. According to their findings (AOR = 3.62, 95% CI: 1.57–8.34, *p* = 0.002), children in schools with these facilities were far more likely to correctly wash their hands than those without (23). Likewise, in Damote Woide District, South Ethiopia, school handwashing facilities were identified as a key factor associated with proper hygiene (AOR = 3.84, 95% CI: 3.60–4.07) (15). These consistent patterns underscore the critical role of supportive institutional infrastructure in enabling routine hygiene behavior. In this study, a notable and statistically significant correlation was found between schools that commemorate Global Handwashing Day (GHD) and the likelihood of students practicing proper hand hygiene in the future. Students attending these schools were more than twice as likely to engage in correct hygiene practices. This observation aligns with research from the Sodo Zuria District, Ethiopia, where GHD celebrations were positively associated with proper hygiene habits (24). Similarly, a study from East Dembiya, Ethiopia, highlighted Global Handwashing Day (GHD) as an important strategy for behavioral communication, as it effectively raises collective awareness and normalizes hygiene practices among peers (14). In Somalia, such advocacy events may provide structured opportunities for hygiene education, collective social modeling, and supply distribution. Therefore, public health interventions should use international advocacy days to establish sustainable institutional commitments to WASH standards. Conversely, a 2021 study in Mogadishu, Somalia, indicated that the primary barrier to handwashing was the lack of dedicated handwashing stations despite the presence of general water sources (13). The consistent observations across the region suggest that placing accessible water stations at points of need is strongly aligned with higher compliance. From a public health perspective, this means that infrastructure investments in the Benadir Region should focus on placing water stations directly outside toilets and cafeteria areas, rather than just ensuring water is available on school grounds. Although these permanent structures were important, the availability of soap and water was not statistically significant. This contrasts with a study in Harar Town, Ethiopia, where access to soap and water was significantly linked to handwashing among primary schoolchildren (AOR = 2.89, 95% CI: 1.39–5.98) (23). This difference might be due to variations in school WASH conditions, supply availability, and hygiene promotion activities. In this study, 67.3% of students reported an absence of soap and water at school, which may have attenuated the observed statistical associations. These findings suggest that the regular availability of soap and water, along with hygiene education and behavioral reinforcement, is crucial for improving handwashing among school children. Identifying parents and teachers as the primary influences on handwashing was significantly linked to better handwashing practices than identifying friends as the primary influence. This aligns with evidence from Ethiopia, which demonstrates that parental and teacher guidance positively correlates with student hygiene compliance. This may be because parents and teachers provide hygiene information, model appropriate behavior, and reinforce proper handwashing through guidance and supervision. Although older students tended to have better handwashing practices, this link was not statistically significant. This might be due to increased health education, awareness, and understanding of hygiene as students advance in school. The strengths of this study include its robust methodology, high statistical power ( N=834 , 100% response rate), and multistage probability sampling design using Probability Proportional to Size allocation across public primary schools in the Benadir Region. Furthermore, the multi-item hygiene index demonstrated strong internal consistency ( ${\text{Cronbach}}^{'}s\;\alpha =0.78$ ), and multivariable logistic regression was used, adjusted for key confounding factors, to identify independent associated factors. However, this study has several limitations must be acknowledged. The cross-sectional design precludes the establishment of temporal causality. Additionally, relying on self-reported face-to-face interviews rather than direct observation or microbial testing introduces potential common method variance, recall bias, and social desirability bias, which may overestimate compliance. Unmeasured institutional variables such as daily water availability and supervision intensity may act as residual confounders. Finally, the findings are restricted to upper primary students (Grades 5–8) in public schools in the Benadir Region and may not be generalizable to private schools, younger pupils, or other regions in Somalia.

## 5 Conclusion

In the Benadir Region’s public primary schools, 76.0% of students in the upper primary grades indicated that they practiced proper handwashing. Multivariable analysis revealed that adherence to proper handwashing was significantly associated with immediate sensory feedback, effective school hygiene facilities, and supportive interpersonal relationships. Notably, immediate sensory satisfaction, such as eliminating unpleasant smells, was more strongly associated with reported handwashing than abstract ideas about disease prevention. Participation in interactive school activities, such as Global Handwashing Day events, was positively linked to compliance. Structurally, having localized handwashing stations at the point of use was associated with an increased likelihood of proper handwashing, highlighting the importance of institutional infrastructure and the continuous availability of soap and water. Additionally, support from parents and teachers was crucial for hygiene compliance. Based on these observations, public health initiatives and educational authorities in Mogadishu should consider strategies that integrate improvements in point-of-use infrastructure, institutionalized hygiene advocacy events, and the active involvement of adult role models to promote sustained hygiene practices among students.

## Data Availability

The original contributions presented in the study are included in the article/supplementary material, further inquiries can be directed to the corresponding author.

## References

[1] De Francisco A Morris J Hall AJ Armstrong Schellenberg JRM Greenwood BM. Risk factors for mortality from acute lower respiratory tract infections in young Gambian children. Int J Epidemiol. (1993) 22:1174–82. doi: 10.1093/ije/22.6.1174, 8144302

[2] Mane AB Reddy NS Reddy P Kv C Nair SS Sriniwas T. Differences of hand hygiene and its correlates among school going children in rural and urban area of Karnataka, India. Arch Med. (2016) 8. doi: 10.21767/1989-5216.1000164

[3] Global Handwashing Partnership. "The handwashing handbook. Washington (DC): global handwashing partnership". In: Prepared by the Global Handwashing Partnership Secretariat. Washington, DC: Global Handwashing Partnership: (2020)

[4] World Health Organization, United Nations Children's Fund (UNICEF). Drinking Water, Sanitation and Hygiene in Schools: Global Baseline Report 2018. New York: United Nations Children's Fund (UNICEF) and World Health Organization (2018).

[5] United Nations Children's Fund (UNICEF). Water, Sanitation and Hygiene (WASH) in Schools. New York (NY): UNICEF Division of Communication (2012).

[6] Dhiraj B Sanjaykr S Aparajita D Preeti PS Amitavakumar DS. Quantification of perception status of hand washing practice among school children in a rural area of West Bengal. Appl Med Sci. (2015) 3:1683–7.

[7] Eshuchi R. Promoting Handwashing with Soap Behaviour in Kenyan Schools: Learning from Puppetry Trials among Primary school children in Kenya. Brisbane, Australia: Queensland University of Technology (2013).

[8] Yallew WW Terefe MW Herchline TE Sharma HR Bitew BD Kifle MW . Assessment of water, sanitation, and hygiene practice and associated factors among people living with HIV/AIDS home based care services in Gondar city, Ethiopia. BMC Public Health. (2012) 12:1057. doi: 10.1186/1471-2458-12-1057, 23216835 PMC3551797

[9] Sekhon H Minhas SA. School based survey on hygiene in a rural area of northern India. Int J Pharma Res Health Sci. (2014) 2:179–84.

[10] WHO Preventing Diarrhoea through Better water, Sanitation and Hygiene: Exposures and Impacts in low- and middle-Income Countries (2014) 1–32. Available online at: http://apps.who.int/iris/bitstream/10665/150112/1/9789241564823_eng.pdf (accessed July 15, 2016)

[11] Nandrup-Bus I. Mandatory handwashing in elementary schools reduces absenteeism due to infectious illness among pupils: a pilot intervention study. Am J Infect Control. (2009) 37:820–6. doi: 10.1016/j.ajic.2009.06.012, 19850374

[12] Federal Democratic Republic of Ethiopia Ministry of Education. National School Water, Sanitation and Hygiene (SWASH) Strategy and Implementation Action Plan. Addis Ababa (Ethiopia): Ministry of Education (2017).

[13] Mohamed MH Ahmed SA. Factors influencing handwashing practice among primary school children attending at Altadamun school, Mogadishu-Somalia. J Health Environ Res. (2021) 7:19–24. doi: 10.11648/j.jher.20210701.14

[14] Engdaw GT Alemu D Alemayehu M Tesfaye AH. Handwashing practices among public primary school students in east Dembiya District, Ethiopia. Am J Trop Med Hyg. (2024) 110:826–34. doi: 10.4269/ajtmh.23-0201, 38471179 PMC10993824

[15] Admasie A Guluma A Debebe A. Hand washing practice and associated factors among primary school children in DamotWoide Woreda of Wolaita zone, South Ethiopia: a cross-sectional study. *Published in Research Square* [Preprint] (2020). doi: 10.21203/rs.3.rs-110596/v1

[16] Besha B Guche H Chare D Amare A Kassahun A Kebede E . Assessment of hand-washing practice and its associated factors among first cycle primary school children in Arba Minch town, Ethiopia, 2015. Epidemiology. (2016) 6:2161–1165. doi: 10.4172/2161-1165.1000247

[17] Mekonnen M Aga F Kinati T Shifera D. Assessment of hand washing practice and associated factors among primary school children in Sebeta town Oromia regional state, Ethiopia. Health Sci J. (2018) 12:544. doi: 10.21767/1791-809X.1000605

[18] Lopez-Quintero C Freeman P Neumark Y. Hand washing among school children in Bogota, Colombia. Am J Public Health. (2009) 99:94–101. doi: 10.2105/AJPH.2007.129759 PMC2636611 19008513

[19] Shrestha A Angolkar M. Impact of health education on the knowledge and practice regarding personal hygiene among primary school children in urban area of Karnataka, India. IOSR J Dent Med Sci. (2014) 13:86–9. doi: 10.9790/0853-13478689

[20] Chen X Ran L Liu Q Hu Q Du X Tan X. Hand hygiene, mask-wearing behaviors and its associated factors during the COVID-19 epidemic: a cross sectional study among primary school students in Wuhan, China. Int J Environ Res Public Health. (2020) 17:2893. doi: 10.3390/ijerph17082893, 32331344 PMC7215913

[21] Alsubaie S Bin Saeed A Al-Shahrani A Al-Subaie F Al-Matham A Al-Kadi A . Hand hygiene knowledge and practices among school children in primary schools in Riyadh, Saudi Arabia. J Infect Public Health. (2016) 9:730–7. doi: 10.1016/j.jiph.2016.04.004

[22] Appiah-Brempong E Appiah-Brempong M. Examining the psychosocial drivers of handwashing behaviour among school children. Health Psychol Behav Med. (2024) 12:2391932. doi: 10.1080/21642850.2024.2391932, 39165273 PMC11334745

[23] Berhanu A Mengistu DA Temesgen LM Mulat S Dirirsa G Alemu FK . Hand washing practice among public primary school children and associated factors in Harar town, eastern Ethiopia: an institution-based cross-sectional study. Front Public Health. (2022) 10:975507. doi: 10.3389/fpubh.2022.975507, 36408055 PMC9670311

[24] Tantu T. Hand washing practice and its determinants among primary school children in Sodo Zuria District, southern Ethiopia. Am J Biomed Sci Res. (2022) 16:694–701. doi: 10.34297/ajbsr.2022.16.002297

